# Correction: An immunological electrospun scaffold for tumor cell killing and healthy tissue regeneration

**DOI:** 10.1039/d5mh90127h

**Published:** 2025-10-14

**Authors:** Xingzhi Liu, Hongbo Zhang, Ruoyu Cheng, Yanzheng Gu, Yin Yin, Zhiyong Sun, Guoqing Pan, Zhongbin Deng, Huilin Yang, Lianfu Deng, Wenguo Cui, Hélder A. Santos, Qin Shi

**Affiliations:** a Department of Orthopedics, The First Affiliated Hospital of Soochow University, Orthopedic Institute, Soochow University 708 Renmin Road Suzhou Jiangsu 215006 P. R. China shiqin@suda.edu.cn; b Shanghai Key Laboratory for Prevention and Treatment of Bone and Joint Diseases, Shanghai Institute of Traumatology and Orthopaedics, Ruijin Hospital, Shanghai Jiao Tong University School of Medicine 197 Ruijin 2nd Road Shanghai 200025 P. R. China wgcui80@hotmail.com; c State Key Laboratory of Molecular Engineering of Polymers, Fudan University No. 220 Handan Road Shanghai 200433 P. R. China; d Animal Experimental Center, Soochow University 99 Renai Road Suzhou Jiangsu 215023 P. R. China; e Department of Pharmaceutical Sciences Laboratory, Åbo Akademi University FI-00520 Finland; f Turku Center for Biotechnology, University of Turku and Åbo Akademi University FI-00520 Finland; g Drug Research Program, Division of Pharmaceutical Chemistry and Technology, Faculty of Pharmacy, University of Helsinki Helsinki FI-00014 Finland; h Helsinki Institute of Life Science (HiLIFE), University of Helsinki Helsinki FI-00014 Finland helder.santos@helsinki.fi; i Department of Medicine, James Graham Brown Cancer Center, University of Louisville 505 South Hancock Street Louisville KY 40202 USA; j Key Laboratory of Stem Cells and Biomedical Materials of Jiangsu Province and Chinese Ministry of Science and Technology 199 Renai Rd Suzhou 215123 China

## Abstract

Correction for ‘An immunological electrospun scaffold for tumor cell killing and healthy tissue regeneration’ by Xingzhi Liu *et al.*, *Mater. Horiz.*, 2018, **5**, 1082–1091, https://doi.org/10.1039/C8MH00704G.

The authors regret errors in Fig. 2c and in Fig. 4c.

**Fig. 2:**
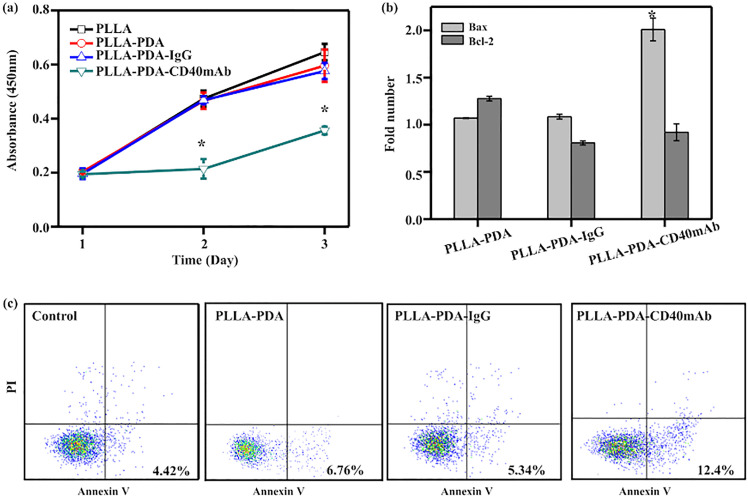
The anti-proliferation effect of CD40mAb released from PLLA-PDA-CD40mAb scaffolds for 24 h towards MDA-MB-231 cells. (a) Cell viability of MDA-MB-231. (b) The relative gene expressions of Bax and Bcl-2 of MDA-MB-231 cells (data are represented as fold changes normalized by cells cultured in cell medium). (c) Flow cytometry assay (FCA) for MDA-MB-231 cell apoptosis. Control: cells seeding onto the plate (compared with the PLLA group; *p < 0.05).

**Fig. 4:**
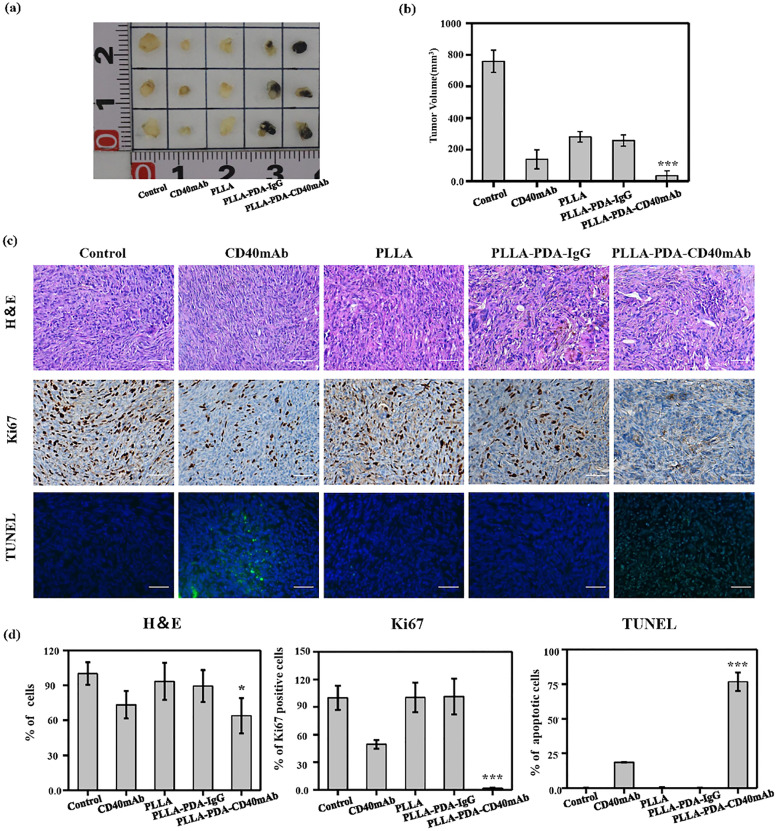
*In vivo* anticancer efficiency of PLLA-PDA-CD40mAb membranes. (a) The representative photographs of the tumors after various treatments and quantification of tumor masses in the different groups indicated. (b) The volumes of tumor masses. (c) H&E stained images, immunohistochemical analysis and TUNEL apoptosis assay (green: apoptotic cells; blue: nuclei) of tumor tissues after membrane-treated therapy. (d) Quantification of the expression of Ki67, TUNEL, H&E assay (***p < 0.001).

In Fig. 2c the incorrect data was used for PLLA-PDA and in Fig. 4c the incorrect image was used for Ki67 PLLA-PDA-IgG.

The corrected figures are shown here.

Independent experts have viewed the corrected figures and the raw data, and they have confirmed that the corrected figures are consistent with the discussions and conclusions presented.

The Royal Society of Chemistry apologises for these errors and any consequent inconvenience to authors and readers.

